# Endoplasmic Reticulum Stress and miRNA Impairment in Aging and Age-Related Diseases

**DOI:** 10.3389/fragi.2021.790702

**Published:** 2022-01-20

**Authors:** Tugce Demirel-Yalciner, Erdi Sozen, Nesrin Kartal Ozer

**Affiliations:** ^1^ Department of Biochemistry, Faculty of Medicine, Marmara University, Maltepe, Turkey; ^2^ Genetic and Metabolic Diseases Research and Investigation Center (GEMHAM), Marmara University, Maltepe, Turkey

**Keywords:** aging, endoplasmic reticulum stress, microRNA, metabolic disorders, neurodegenerative diseases, cardiovascular diseases, cancer

## Abstract

Aging is a physiological process defined by decreased cellular and tissue functions. Reduced capacity of protein degradation is one of the important hallmarks of aging that may lead to misfolded protein accumulation and progressive loss of function in organ systems. Recognition of unfolded/misfolded protein aggregates *via* endoplasmic reticulum (ER) stress sensors activates an adaptive mechanism, the unfolded protein response (UPR). The initial step of UPR is defined by chaperone enhancement, ribosomal translation suppression, and misfolded protein degradation, while prolonged ER stress triggers apoptosis. MicroRNAs (miRNAs) are non-coding RNAs affecting various signaling pathways through degradation or translational inhibition of targeted mRNAs. Therefore, UPR and miRNA impairment in aging and age-related diseases is implicated in various studies. This review will highlight the recent insights in ER stress–miRNAs alterations during aging and age-related diseases, including metabolic, cardiovascular, and neurodegenerative diseases and several cancers.

**GRAPHICAL ABSTRACT:**
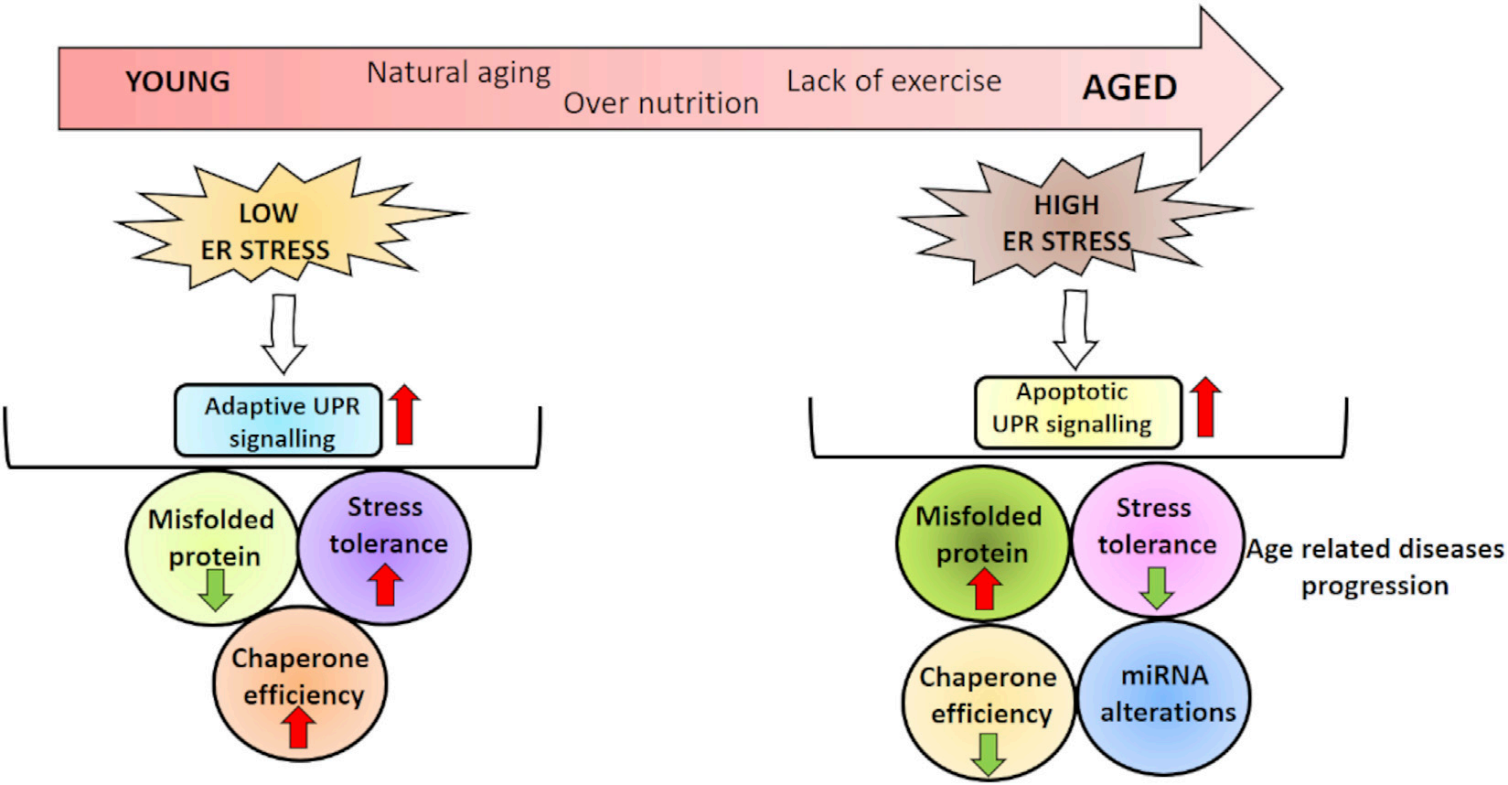


## Introduction

Aging is a process that results in decreased body homeostasis and increased risk of disease or death (Ito and Barnes, 2009). Mainly, nine hallmarks in aging have been described as follows: 1) telomere attrition, 2) epigenetic alterations, 3) genomic instability, 4) mitochondrial dysfunction, 5) deregulated nutrient sensing, 6) cellular senescence, 7) stem cell exhaustion, 8) altered intercellular communication, and 9) loss of proteostasis (Lopez-Otin et al., 2013). In recent years, metabolic disorders, including obesity, cardiovascular diseases (CVDs), and neurodegenerative disorders, have also been described as other hallmarks in the aging process (Spinelli et al., 2020).

Recognition of unfolded/misfolded proteins *via* endoplasmic reticulum (ER) chaperones is a well-studied process, stimulating unfolded protein response (UPR). UPR is an adaptive mechanism against ER stress, involving three signaling pathways organized *via* transmembrane sensors. Activation of these sensors influences various parameters that affect protein metabolism, redox homeostasis, and apoptosis. Evidence has implicated the crucial role of protein toxicity caused by aging-related UPR activity decline in age-related disorders. In this context, many studies have determined that ER chaperones (PDI, GRP78, and GRP94) and UPR signaling are decreased in aging cells (Naidoo et al., 2008).

MicroRNAs (miRNAs) are non-coding RNAs regulating the existence of their target genes through inhibition of translation or induction of mRNA degradation. miRNAs control gene expression by targeting hundreds of mRNAs. Lin-4, the first discovered miRNA in *Caenorhabditis elegans*, is an essential gene involved in all larval stages (Lee et al., 1993). Until now, about 2,500 miRNAs are determined in mammals and are still being discovered (O'Brien et al., 2018). Although miRNAs have a crucial role in tissue homeostasis, they also have function in metabolic and neurodegenerative diseases and CVDs (Rupaimoole and Slack, 2017). A number of miRNAs were also demonstrated to modulate the major components in UPR signaling (Byrd and Brewer, 2013). Although it is clear that ER stress and UPR activation are components of the senescent phenotype, it has not yet been fully elucidated whether ER stress/UPR is the cause or consequence of cell senescence and its relationship with miRNA in aging. The first part of our review will discuss the general principles of ER stress response as well as its interaction with miRNA and aging. In the second part, ER stress response and miRNA impairment in age-related diseases, such as metabolic disorders, neurodegenerative diseases, CVD, and cancer, will be discussed.

## The Endoplasmic Reticulum Stress Response

**FIGURE 1:**
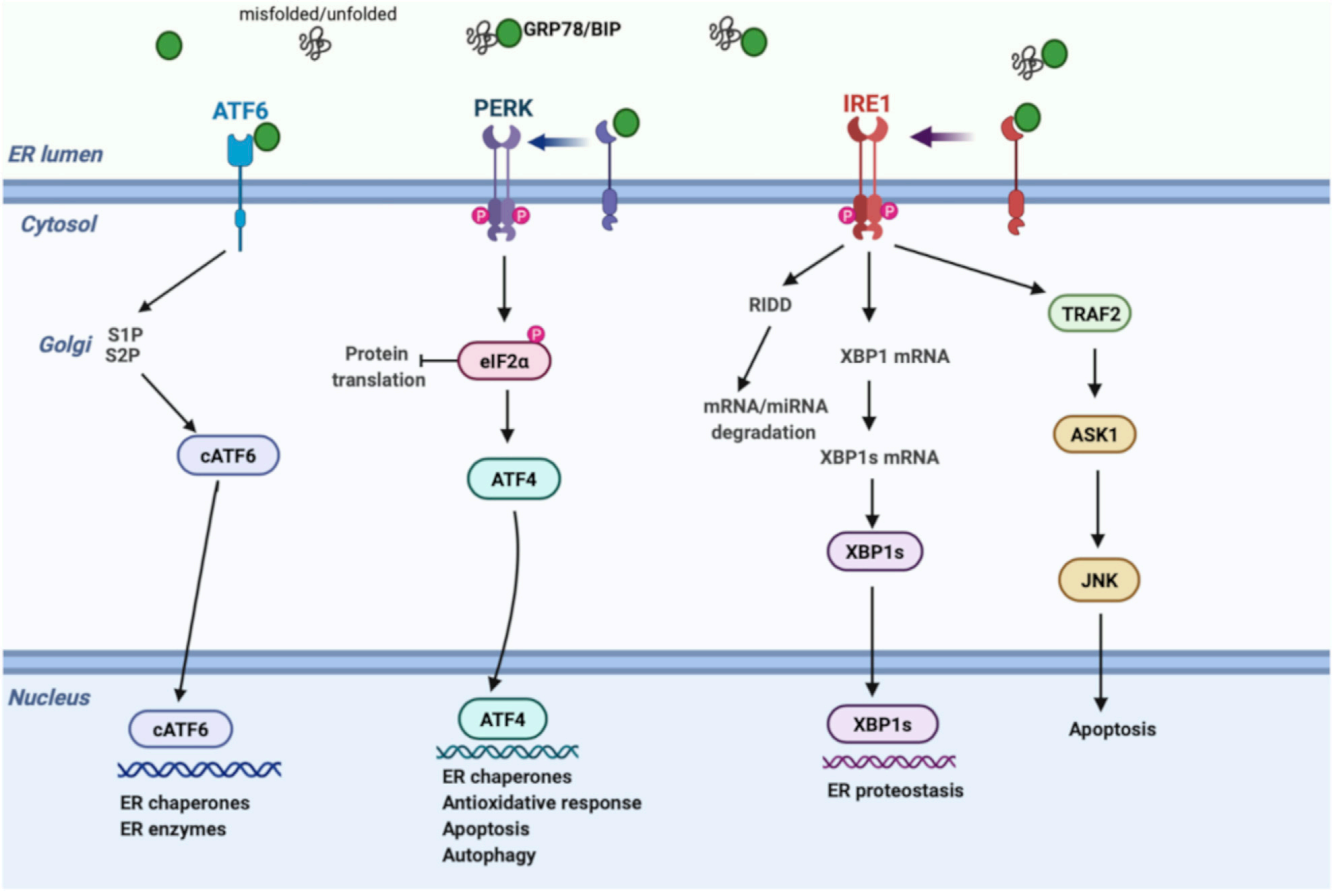
Schematic model of UPR signalling. Under ER stress, GRP78 is released from IRE1, PERK, and ATF6, inducing three separate UPR signals; (1) PERK phosphorylates eIF2α, inhibiting protein translation. Phospho eIF2α enhances ATF4 expression, activating the transcription of UPR genes that regulate the antioxidative response, autophagy, and apoptosis mechanisms. (2) IRE1 induces XBP-1 splicing and RIDD pathway through RNase activity. XBP1s regulates genes involved in ER proteostasis, while RIDD modulates degradation of mRNAs and miRNAs to reduce protein burden. On the other hand, cytosolic domain of IRE1 interacts with TRAF2 to activate ASK1 and JNK, inducing apoptosis. (3) ATF6 is transported to the Golgi apparatus followed by its cleavage *via* proteases. Then, active cATF6 enters the nucleus and modulates ER enzymes and chaperones. ASK1, apoptosis signal-regulating kinase; ATF6, activating transcription factor 6; ATF4, activating transcription factor 4; cATF6, cleaved ATF6; eIF2α, eukaryotic initiation factor 2α; GRP78, glucose-regulated protein 78; IRE1, inositol-requiring enzyme; JNK, c-Jun amino-terminal kinase; PERK, protein kinase-like ER kinase; RIDD, regulated IRE1-dependent decay; S1P, site 1 protease; S2P, site 2 protease; TRAF2, tumor necrosis factor receptor-associated factor 2; UPR, unfolded protein response; XBP1, X-box binding protein 1; XBP1s, spliced x-box binding protein 1.

The ER is a membrane-bound organelle involved in protein folding, calcium homeostasis, and lipid biosynthesis. Under normal conditions, chaperones and other proteins including GRP78, GRP94, the lectins, calnexin, calreticulin, and foldases have a central effect in the ER in performing these functions. In a variety of pathophysiological conditions, aberrant accumulation of unfolded/misfolded proteins in the ER results in ER stress and triggers the UPR pathway to degrade unfolded/misfolded proteins, suppress protein synthesis, and increase the folding ability. UPR regulation is achieved *via* three transmembrane proteins: 1) protein kinase-like ER kinase (PERK), 2) inositol-requiring enzyme 1 (IRE1), and 3) activating transcription factor 6 (ATF6) (Figure 1). In homeostatic conditions, GRP78 suppresses the induction of IRE1, PERK, and ATF6 by binding their luminal surface. Once ER stress is induced, IRE1, PERK, and ATF6 disassociate from GRP78, enhancing unique signaling pathways (Sozen and Ozer, 2017).

As a quick and first response against ER stress, PERK reduces protein synthesis by effecting translation in mammalian cells. Mechanistically, activated PERK phosphorylates eIF2α, leading to ribosome inhibition and brief attenuation of global cell translation. During prolonged stress conditions, phosphorylated eIF2α selectively stimulates ATF4, upregulating transcription of a gene encoding pro-apoptotic CHOP and GADD34. CHOP upregulates pro-apoptotic genes, while GADD34 controls protein synthesis by eIF2α dephosphorylation. Therefore, besides its effect on pro-survival signaling as an initial response, PERK also induces pro-apoptotic mechanism through upregulation of CHOP and GADD34 under prolonged ER stress (Sozen et al., 2015).

IRE1 signaling is the second most important branch of the UPR pathway. Today, it is well established that IRE1 has dual effects through its endoribonuclease and kinase domains. To restore proteostasis, cytosolic endoribonuclease domain initiates two distinct events: 1) activation of regulated IRE1-dependent decay (RIDD) signaling and 2) splicing of XBP1 mRNA (Ron and Walter, 2007). RIDD pathway enhances degradation of mRNAs and prevents newly synthesized proteins from entering the ER, while XBP1 splicing upregulates chaperone gene expression. Unconventional splicing of XBP1 mRNA into XBP1s (spliced form) also interacts with certain proteins in metabolism such as FOXO1 (Zhou et al., 2011) and PI3K (Park et al., 2010). In addition, serine/threonine kinase domain of active IRE1 interacts with TRAF2 to activate ASK1, inducing apoptotic signals, including JNK and p38 (Darling and Cook, 2014).

ATF6, the third branch of the UPR, is mainly involved in expanding the functional capacity of the ER during UPR. After ER stress is induced, ATF6 is transported to the Golgi by coat protein complex 2 and cleaved by site-1 and site-2 proteases. Cleaved active ATF6 then enters the nucleus and modulates ER chaperones and enzyme levels, such as PDI, GRP78, and GRP94 (Westrate et al., 2020).

### Endoplasmic Reticulum Stress and MicroRNA

miRNAs are single-stranded non-coding RNAs that modulate gene activity by controlling mRNA degradation/translation. Transcription of pri-miRNA from DNA is the initial phase in miRNA biogenesis followed by the formation of pre-miRNA and mature miRNA (O'Brien et al., 2018). Often, miRNAs cause degradation or translational inhibition of mRNAs upon recognition in the 3′ untranslated region (3′ UTR) (Broughton et al., 2016). However, in certain cases miRNAs are determined to activate mRNA expression (Vasudevan, 2012). Changes in miRNA expressions take a critical role in several biological mechanisms and human diseases (Paul et al., 2018). Additionally, miRNAs secreted into mammalian body fluids have been accepted as possible markers for various human diseases (Wang et al., 2016; Huang, 2017; Siracusa et al., 2018; He et al., 2020).

**FIGURE 2:**
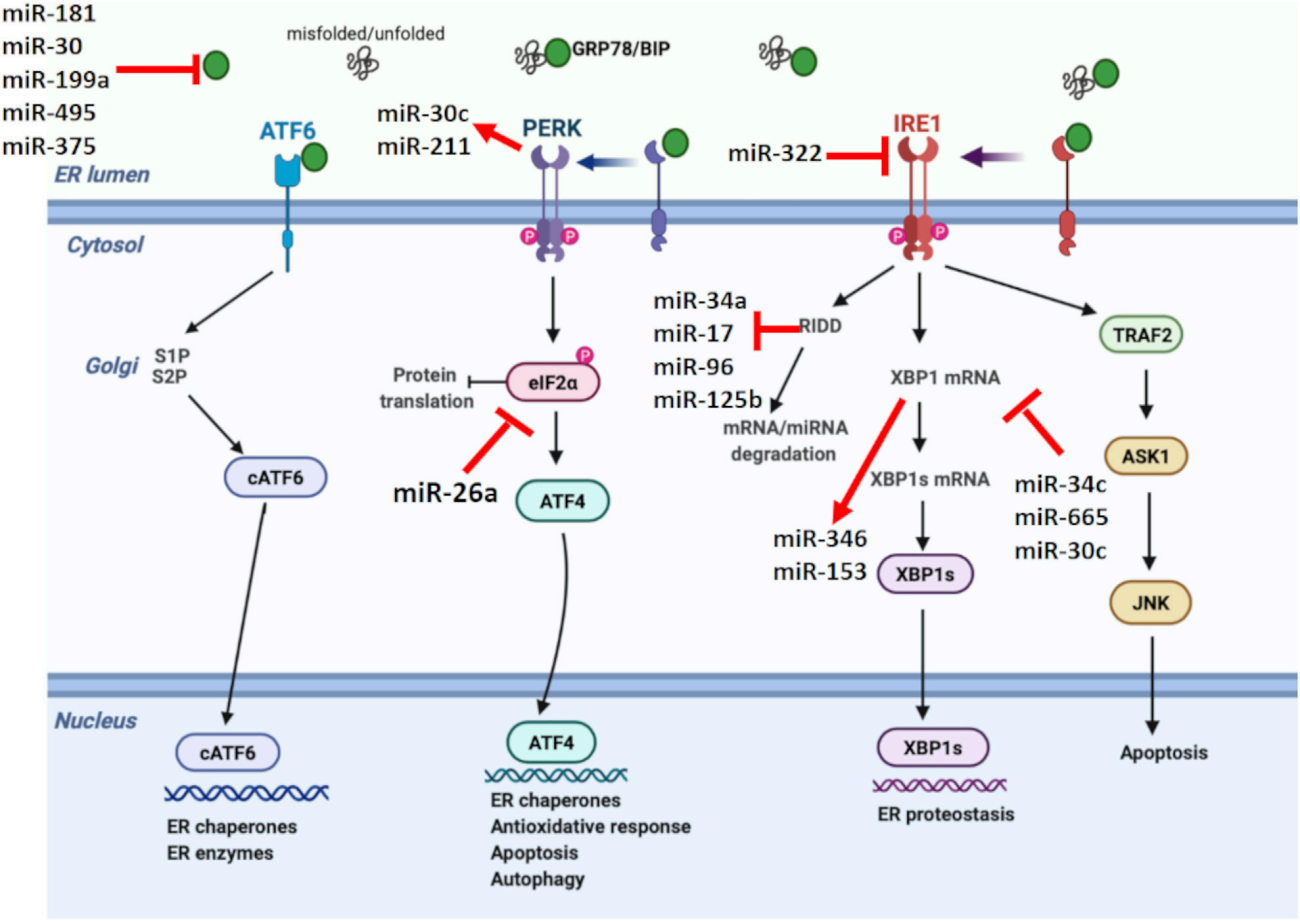
Unfolded protein response signaling with their regulating microRNAs. miRNAs have a crucial role in shaping the UPR, while miRNA expression is also regulated by UPR. miR-181, miR-30, miR-199a, miR-495, and miR-375 negatively regulates GRP78. On the other hand, miR-322 suppresses IRE1. XBP1 and RIDD signaling, members of IRE1 branch in UPR, regulate miR-153, miR-346, miR-34a, miR-17, miR-96, and miR-125b. Additionally, miR-34c, miR-665, and miR-30c are known to target XBP1, while miR-26a suppresses eIF2α, reducing the protein translation. Certain miRNAs also form a link between UPR branches. In this direction, PERK-mediated miR-30c activation inhibits XBP1 pathway and establishes a negative crosstalk between PERK and IRE1 branches of UPR.

Related evidence in mammalian cells suggests that miRNAs are involved in shaping the ER stress, while miRNA expression is also regulated by ER stress (Figure 2) (Mesitov et al., 2017). Several miRNAs such as miR-181 family, miR-30 family, miR-199a, miR-495, and miR-375 are reported to target GRP78 mRNA (Kim and Croce, 2021). In addition, it was discovered that repression of PDIA6, an activator of IRE1, by miR-322 indirectly inhibits IRE1 expression (Groenendyk et al., 2014). Certain miRNAs such as miR-34c, miR-665, and miR-30c are known to directly target XBP1 mRNA, while miR-346 and miR-153 are controlled by XBP1 during ER stress (Byrd et al., 2012; Li M. et al., 2017; Bartoszewska et al., 2019). Besides miRNAs targeting UPR parameters, a couple of miRNAs are also regulated by UPR. Behrman and Walter (Behrman et al., 2011) showed that miR-711, miR-2137, miR-1897, miR-689, miR-708, miR-712, miR-762, and miR-2132 were increased by ER stress, while miR-322, miR-503, and miR-351 were inhibited. Another study showed PERK-mediated miR-211 increase in NIH3T3 fibroblast cells, reducing apoptosis *via* CHOP inhibition (Chitnis et al., 2012).

RIDD is another pathway in which miRNA expression is modulated by ER stress (Zhang et al., 2020). Under prolonged stress conditions, RIDD degrades pre-miRNAs as well as pro-survival protein encoding mRNAs. IRE1 processes pre-miR-34 and pre-miR-200, resulting in their degradation *via* RIDD signaling (Wang et al., 2018). IRE1-mediated RIDD activation is also reported to cleave the anti-apoptotic miR-34a, miR-17, miR-96, and miR-125b precursors, reducing caspase 2 expression (Upton et al., 2012). Additionally, miRNAs have the ability to form a link between different UPR branches by regulating certain UPR parameters. For example, PERK-mediated miR-30c activation inhibits XBP1 pathway and establishes a negative crosstalk between IRE1 and PERK branches. Therefore, prolonged PERK activation is assumed to inhibit the pro-survival mechanism of UPR by downregulating IRE1-XBP1 signaling (Byrd et al., 2012). The regulatory role of miR-30c between UPR branches was also demonstrated in ovarian malignant cells by inducing apoptosis through CHOP activation as well as XBP1 suppression (Rezghi Barez et al., 2021).

### Endoplasmic Reticulum Stress and Aging

Aging is a complex process that impacts at the cellular and tissue levels, increasing the risk of disease/death (Gems and Partridge, 2013; Cohen, 2018). It is characterized by physiological alterations in body composition, including decreases in sex steroids and growth hormone (Barzilai et al., 2012). However, well-established hallmarks of aging are associated with several conditions that induce ER stress, including increased oxidative stress, deficiencies in mitochondrial and autophagic functions, disruptions in Ca^2+^ homeostasis, disorders in protein homeostasis, and energy metabolism.

Several studies have shown reduced ER chaperone levels and UPR signaling in different tissues from aged model organism. In a study, rats were divided into four groups: suckling (0–4 day old), young (1 month old), adult (>6 months old), and old (>18 months old). ATF4, GRP78, and eIF2α protein expressions were evaluated and found to be decreased with age in hippocampus tissue (Hussain and Ramaiah, 2007). In accordance, GRP78, PDI, and calnexin protein expressions were downregulated, while proapoptotic caspase 12 and CHOP protein expressions were upregulated in hippocampus tissues of aged rats (23–26 months) compared to young (4–6 months) (Paz Gavilan et al., 2006). In addition to decreased protein folding capacity and increased proteotoxicity, accumulation of oxidized chaperones in ER was also reported in aged C57BL/6J mice (20–24 months) (Nuss et al., 2008). Another study showed decreased eIF2α amount in cerebral cortex of C57BL/6J male mice aged 22–24 months (Naidoo et al., 2008). Li and Holbrook (2004) investigated ER stress in hepatocytes from young (4–5 months) and old (24–26 months) rats and reported a decrease in eIF2α expression with aging. However, some studies have reported an increase in GRP78 gene expression in the heart and kidney of aged C57BL/6 mice (24 months) (Takeda et al., 2013; Sreedhar et al., 2016). Rabek et al. (2003) determined that GRP78, calreticulin, and PDI exhibited an age-related induction in liver tissue of 24-month-old mice. Ghosh et al. (2015) observed elevated GRP78, CHOP, cleaved ATF6, IRE1, and XBP-1 levels in adipose tissue stromal cells of old mice (18–20 months) compared to young (4–6 months). Furthermore, other researchers have suggested that GRP78 levels are not changed in various tissues from diabetic kidney of 22-month-old C57BL/6 mice (Wu et al., 2010; Naidoo et al., 2011; Jiao et al., 2012; Torres-Gonzalez et al., 2012) as well as skeletal muscle of 29-month-old rats (Baehr et al., 2016). While there was no change in GRP78 levels in aged *Drosophila melanogaster* (8 weeks), a decrease in eIF2α expression was determined (Brown et al., 2014). In another study, GRP78, ATF6, and IRE1 expressions were determined to not changed in 70-year-old human muscle tissue (Ogborn et al., 2014). In addition, apoptosis and ER stress were evaluated in macrophages isolated from young (6–8 weeks) and old (16–18 months) mice, and aged macrophages were found to trigger apoptosis by inducing IRE1 activation (Song et al., 2013). Takeda et al. (2013) determined that ATF6 was increased in kidney tissue of 24-month-old mice.

As a result, studies conducted so far have demonstrated that ER stress may be affected differently during aging. The difference in ER stress parameters might be thought to depend on the model organism used in the study or the tissue examined. However, most of the findings suggest that many components of the UPR and response to stress factors decrease with age, leading to diseases that cause or exacerbate existing diseases.

### Aging and MicroRNA

miRNAs, effective players in regulating many different processes, are reported as significantly increased or decreased in aging. In *Caenorhabditis elegans*, miR-34 and miR-71 were found to be increased during aging, whereas miR-34 was the main contributor in the regulation of cellular senescence (Smith-Vikos and Slack, 2012). In addition, *in vivo* studies have demonstrated miRNA alterations in tissues such as liver, brain, muscle, and heart during aging. Maes et al. (2008) reported increased miR-93 and miR-214 levels in 33-month-old mice liver compared to 4- to 10-month-old mice liver. Another study using rat also showed an induction in miR-34a and miR-93 expressions with aging process in the liver (Li et al., 2011b). Besides the liver, miRNA alterations during aging are also reported in brain tissues of animals. In a study using brain tissues of young, adult, and aged rats, sequencing was performed to determine the miRNA profiles and found 171 potential miRNAs in addition to the 547 miRNAs differentially expressed between groups (Yin et al., 2015). Additionally, expressions of miR-22, miR-101a, miR-720, and miR-721 were upregulated with aging in mouse brain (Li et al., 2011a). In skeletal muscle, miR-468, miR-7, miR-542, and miR-698 were found to be upregulated with aging, while miR-382, miR-124a, miR-181a, miR-434, miR-221, and miR-455 were downregulated (Hamrick et al., 2010). miR-21 and miR-22 levels were also induced in heart tissue of aged mice compared to younger ones (Zhang et al., 2012; Jazbutyte et al., 2013).

Related studies using various peripheral fluids, including plasma, serum, and saliva, in young and aged individuals have determined altered miRNA expressions. Noren Hooten et al. (2013) found that miR-181a, miR-1248, and miR-151a in the serum of the elderly (mean age 64.6 years) were significantly downregulated compared to those in younger individuals (mean age 30 years). Additionally, serum samples of Chinese individuals were collected from four different groups with mean ages of 22, 40, 59, and 70 years, and miR-29b, miR-106b, miR-130b, miR-142, and miR-340 levels were found to decrease with age (Zhang et al., 2015). In a study conducted with 374 young and elderly populations, plasma levels of miR-126, miR-30c, miR-30b, miR-210, and miR-142 were increased with age, while miR-93 was decreased (Ameling et al., 2015). Similarly, miR-24 levels in saliva were found to be increased in a mean age of 66 years compared to mean age of 21 years (Machida et al., 2015).

Besides these studies, it is important to examine centenarians as they exhibit a successful example of aging. There are a limited number of publications examining miRNA expressions in centenarians. miRNA analysis in centenarians was first reported in mononuclear cells and showed that 15,644 miRNA expressions in centenarians significantly overlapped with those in young individuals but not in octogenarians (Serna et al., 2012). The same group published another study, and they showed that RNA Pol II, Dicer, Drosha, and Exportin 5 activities, crucial players in miRNA biogenesis, were upregulated in centenarians compared to octogenarians (Borras et al., 2017). Additionally, Gombar et al. (2012) evaluated the miRNAs in B cells of Ashkenazi Jewish centenarians by sequencing and determined 22 increased miRNAs in addition to 2 reduced miRNAs over centenarians compared to mean age of 50 years. Beside those findings, miR-21, miR-425, and miR-212 were shown to be reduced in centenarians compared to those with an age between 30 and 50 years (Balzano et al., 2017). Collectively, numerous studies greatly expanded our understanding of the effect of miRNAs during aging. Although these findings indicate miRNA alterations, additional studies are needed to inspire the use of miRNAs therapeutically.

### Aging, Endoplasmic Reticulum Stress, and MicroRNA

Involvement of miRNAs in regulating stress-induced responses (Ong, et al., 2019) and the role of UPR in controlling proteostasis in aging models (Martinez et al., 2017) are reported in a variety of studies. Besides the theories and findings about the different roles of ER stress and miRNA in aging, the limited number of studies discovering ER stress and miRNA impairment in aging makes it difficult to make a definitive judgement. Therefore, the question arises whether the ER stress–miRNA relationship is involved in the initiation or maintenance of aging characteristics. Due to the limited information on this issue, the potential effect of ER stress and miRNA on aging as well as possible interaction points will be discussed in this part. IRE1-mediated RIDD activation might be an important process in this context. RIDD activation is determined in ER stress-mediated degradation of various miRNA families, including miR-34, miR-200, miR-17, miR-96, and miR-125b (Upton et al., 2012; Wang et al., 2018). In drosophila, miRNA-mediated alterations in chaperone proteins are reported to have a crucial role in brain aging. miRNA-34 increase determined in this study is reported to be an essential process required for healthy brain aging in drosophila. Researchers have also showed that miR-34 modulates the chaperone system and prevents neurodegeneration by inhibiting polycomb repressor complex 2 in aged drosophila brain (Kennerdell et al., 2018). However, they did not indicate the role of RIDD in this process. We believe it would be beneficial to investigate miR-34 and other miRNAs in aging by associating with RIDD. Additionally, miRNAs that mediate a link between different branches of UPR should be evaluated during aging. In this context, miR-181 family, miR-30 family, miR-199a, miR-495, miR-375, miR-322, miR-34c, miR-665, miR-346, miR-153, and miR-211 might provide useful information in encouraging miRNA-mediated silencing therapy as a beneficial strategy against ER stress-induced disease development.

Compared to the aging process, the effect of miRNA-ER stress in age-related diseases has been more investigated. As stated in the second part of our review, multiple studies have revealed ER stress–miRNA interaction in metabolic disorders, neurodegenerative diseases, and CVDs and cancer in many aspects. We believe that studying these aspects of age-related diseases during the aging process will be important in providing new perspectives. In summary, although many lines of evidence support a strong connection between aging, UPR, and miRNA, experiments are further needed to better characterize molecular and functional links between these, especially in *in vitro* and *in vivo* models in which data are very few presently. A comprehensive understanding of the relationship between aging miRNA and ER stress is thought to help improve this universal process that compromises our quality of life.

## Endoplasmic Reticulum Stress and MicroRNA Alteration in Age-Related Diseases

**FIGURE 3:**
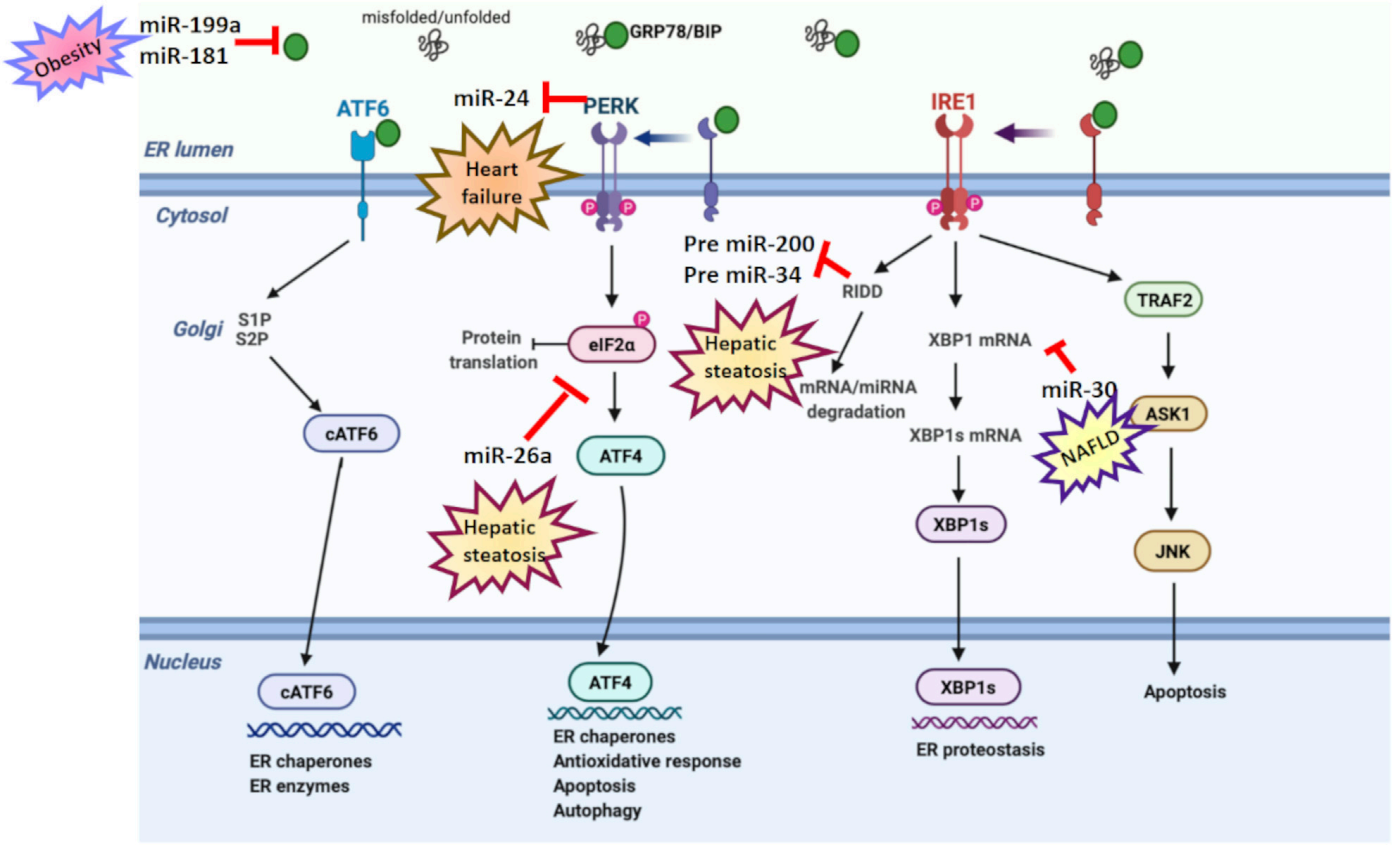
ER stress and microRNA relation in age-related diseases. Various studies have reported a dual relation between miRNA and ER stress in age-related disorders. miR-199a and miR-181 suppress GRP78 expression in obesity. miR-30 downregulates XBP-1 expression in NAFLD. miR-26a reduces eIF2α in hepatic steatosis. However, PERK suppresses miR-24 in heart failure, while RIDD activation degrades pre-miR-200 and pre-miR-34 in hepatic steatosis.

Studies have identified miRNAs as key regulators of many genes involved in cellular signaling pathways (Wang Y. et al., 2008). As shown in Tables 1 and 2, various *in vitro* and *in vivo* studies have reported a dual relationship between miRNA and ER stress in age-related diseases. In addition, the relation between ER stress and miRNA in age-related diseases is shown in Figure 3. In this part of our review, we will highlight ER stress and miRNA impairment in age-related diseases, including metabolic disorders, neurodegenerative diseases, and CVDs as well as cancer.

**TABLE 1:** MicroRNA-mediated ER stress regulation during age-related diseases.

miRNA	ER stress	Age-related diseases
miR-181a	Suppresses GRP78 expression	Obesity (Mesitov et al., 2017)
miR-199a		
miR-30c	Reduces ER stress	Obesity (Menikdiwela et al., 2019)
miR-708	Induces ER stress	Obesity (Menikdiwela et al., 2019)
miR-143		
miR-34a	Interacts with ER stress	NAFLD (Gjorgjieva et al., 2019)
miR-122	Decreases UPR-mediated apoptosis	NAFLD (Gjorgjieva et al., 2019)
miR-30	Downregulates of XBP-1 expression	NAFLD (Gjorgjieva et al., 2019)
miR-26a	Targets the eukaryotic initiation factor 2α and decrease ER stress and hepatic steatosis	Hepatic steatosis (Xu et al., 2021)
miR-99b	Induces ER stress	Alzheimer's disease (Ye at al., 2015)
miR-100		
miR-16-1	Inhibits Hsp70 and promotes α-synuclein aggregation	Parkinson's disease (Zhang and Cheng, 2014)
miR-7	Reduces ER stress	Parkinson's disease (Shen et al., 2021)
miR-29a	Induces ER stress	Amyloid lateral sclerosis (Nolan et al., 2014)
miR-103	Induces ER stress	Atherosclerosis (Jiang et al., 2020)
miR-451-a	Induces ER stress	Colorectal cancer (Xu et al., 2019)
miR-663	Induces ER stress	Hepatocellular carcinoma (Huang et al., 2016)

**TABLE 2:** ER stress-mediated miRNA regulation during age-related diseases.

ER stress	miRNA	Functions
IRE1α inhibition	Induces miR-34a	Protective effects against Aβ-induced injury in SH-SY5Y cells (Li et al., 2019)
PERK	Suppresses miR-24	Protection against mitochondrial dysfunction in heart failure (Shimizu et al., 2020)
UPR activation	Inhibits mir 199a and mir214	Regulates tumor survival and progression (Duan et al., 2012)
RIDD activation	Degrades pre-miR-200 and pre-miR-34	Prevents hepatic steatosis (Wang et al., 2018)

### Metabolic Disorders

Metabolic disorders generate serious threats to human health and lead to severe chronic diseases such as obesity, diabetes, and fatty liver (Lemmer et al., 2021). While the risk of developing metabolic disorders increases with age, the aging process is accelerated in the presence of these diseases (Guarner-Lans et al., 2011). Obesity, insulin resistance, inflammation, and hypertension, which increase in prevalence during aging, also contribute to metabolic syndrome (Diaz et al., 2019).

A number of diseases, including metabolic disorders, have been found to be associated with ER abnormalities since ER is the major organelle in regulating calcium signaling, protein and lipid metabolism, and gluconeogenesis. Obesity and insulin resistance, two major metabolic disorders occurring all around the world, are also related to ER stress (Han et al., 2006; Hotamisligil, 2006). JNK and NFκB pathways are principal inflammatory signals linked to disturbance in insulin action (Deng et al., 2004). IRE1 is associated with TRAF2-mediated activation of JNK as described above (Sozen and Ozer, 2017), while NFκB signaling is mediated with activation of both IRE1 and PERK, through different mechanisms. IRE1-mediated IKK induction through TRAF2 activation triggers NFκB signaling and stimulates inflammatory genes (Salminen et al., 2020). PERK-mediated eIF2α kinase also stimulates NFκB signaling (Jiang et al., 2003). Insulin-receptor substrate 1 (IRS1), activated by phosphorylation of insulin receptors, is crucial in the distribution and modulation of insulin signaling. ER stress has also been demonstrated to reduce insulin receptor signaling by activating JNK and induce insulin resistance (Koh et al., 2013; Brown et al., 2020). IRS1 was suppressed by ER stress-mediated JNK hyperactivation in the liver tissue of obese mice (Ozcan et al., 2004). The capacity of ER stress in disturbing IRS1 induction is also reported in brain tissue of obese rats (Liang et al., 2015).

Expression of miRNAs is differentially modulated in insulin-sensitive tissues during metabolic disorders, including type 2 diabetes (Menikdiwela et al., 2021). In this scope, it has been shown that the regulation of UPR branches is carried out by a couple of miRNAs. GRP78 expression has been determined to be suppressed by miR-181a and miR-199a (Mesitov et al., 2017). Menikdiwela et al. (2019) also showed ER stress signaling is reduced by miR-30c, while it is induced by miR-708 and miR-143 in obese mice. Further, miRNAs have been known to have a crucial effect in fatty liver diseases (Bozaykut et al., 2016). However, there are limited studies examining ER stress–miRNA relationship in hepatocytes (Xu et al., 2021). miR-34a, miR-122, and miR-30 were determined to enhance the development and progression of nonalcoholic fatty liver disease (NAFLD) *via* interacting with ER stress (Gjorgjieva et al., 2019). miR-122-mediated UPR downregulation resulted in reduced apoptosis in human hepatoma cells (Yang et al., 2011), while miR-34a-increased GRP78 levels activated ER stress in mouse liver (Stacchiotti et al., 2019). RIDD-mediated pre-miR-200 and pre-miR-34 degradation are involved in hepatic steatosis (Wang et al., 2018), while miR-23a-mediated ER stress activation supported hepatocarcinogenesis (Liu et al., 2019). miR-26a is other miRNA that was found to reduce ER stress and hepatic steatosis in murine primary hepatocytes, human hepatoma cells, and mice fed a high-fat diet. Mechanistically, miR-26a directly targets eIF2α, reducing the protein burden by inhibiting translation (Xu et al., 2021).

### Neurodegenerative Diseases

A certain feature of neurodegenerative diseases is the formation of misfolded protein aggregates that affect neuronal death. These diseases, including Alzheimer's disease (AD), Parkinson's disease (PD), amyloid lateral sclerosis (ALS), and Huntington's, are associated with aging processes that normally occur later in life (Lindholm et al., 2006). Given the prevalence in the elderly population, brain aging may form a continuum with neurodegeneration (Santulli et al., 2015). AD and PD prevalence is high in the elderly population, and also, the risk of developing these diseases enhances by age (Hou et al., 2019). Recent studies determined the protective effect of initial UPR steps, while prolonged activation induced apoptotic signaling (Perri et al., 2015). It has been determined that UPR parameters, including GRP78, IRE1, ATF6, PERK, eIF2α, and CHOP, are increased in patients with AD (Garcia-Gonzalez et al., 2018), PD (Gerakis and Hetz, 2018), ALS (Prell et al., 2019), and prion diseases (Lindholm et al., 2017).

AD is characterized by accumulation of amyloid-β (Aβ) plaques that are mediated by β-secretase (BACE) and γ-secretase from cleavage of amyloid β-precursor protein (APP) (Jiang et al., 2012). Association between ER stress and synthesis of Aβ, BACE, and γ-secretases has been well identified (Walter and Ron, 2011). In this context, UPR activation upregulated PERK, eIF2α, and IRE1 levels in the temporal cortex and hippocampus (Xiang et al., 2017). In primary rat embryo neuronal cultures, UPR also induced the activity of glycogen synthase kinase 3p, leading to tau phosphorylation (Resende et al., 2008).

PD is another neurodegenerative disease defined by enhanced dopaminergic neuron loss and formation of modified α-synuclein aggregates with a prevalence increasing with age (de Lau and Breteler, 2006). Numerous studies have reported ER stress in PD pathogenesis and determined its role in α-synuclein accumulation in PD patients (Hoozemans et al., 2007). ATF6 deficiency enhanced the dopaminergic neuron damage, triggering PD development in mice (Egawa et al., 2011). *In vivo* studies have also demonstrated the capacity of IRE1-XBP1 signaling in controlling dopaminergic neuron survival (Valdes et al., 2014).

In mammals, more miRNAs are expressed in the brain than in many other tissues, and thus miRNAs play key functions in neuronal development and in the pathogenesis of neurodegenerative diseases (Maciotta et al., 2013; Rajgor and Hanley, 2016). Related studies determined abnormal miRNA expression in brains of AD and suggested the involvement of miRNA alteration in tau phosphorylation or Aβ deposition (Wu et al., 2016). miR-101 modulated APP expression in rat hippocampal neurons (Vilardo et al., 2010), while miRNA-107, which targets BACE1, was lower in AD patients (Wang W.-X. et al., 2008). Another study with middle-aged rats determined that miR-17, miR-106b, miR-153, and miR-101 expressions, which target APP, were reduced in brain tissue (Che et al., 2014). Decreased miR-186 levels in the cortex of aged mice were also found to be related to BACE1 expression (Kim et al., 2016). A study with AD patients showed the downregulated miRNA-29a/b-1 expression in the cortex, associated with BACE1 protein (Hebert et al., 2008). miR-141, miR-200a/b/c, and miR-429 were found to be increased in early-age AD in mice (Wu et al., 2016). However, a number of researches revealed possible interaction between ER stress and miRNA alterations during AD pathogenesis. For example, ER stress inhibits PTEN expression by inducing miR-200c to protect neurons against Aβ toxicity in the initial step of Aβ damage (Wu et al., 2016). Ye et al. showed that miR-99b and miR-100 induced ER stress in AD mice (Ye et al., 2015). On the other hand, miR-34a upregulation in SH-SY5Y cells has been reported to be protective against Aβ-mediated injury by causing IRE1 inhibition (Li et al., 2019).

In addition, various miRNAs involved in the pathogenesis of PD have been identified. Decreased miR-34c and miR-34b expression in SH-SY5Y cells were demonstrated to contribute to PD pathogenesis by increasing α-synuclein (Kabaria et al., 2015). Zhang and Cheng (2014) determined the contribution of miR-16-1 to PD development by inhibiting Hsp70 levels and inducing α-synuclein accumulations in SH-SY5Y cells. On the contrary, miR-7-mediated ER stress suppression in SH-SY5Y has been determined to be protective against PD (Shen et al., 2021).

### Cardiovascular Diseases

Aging is another important risk factor in CVDs besides the obesity and diabetes. The prevalence of CVDs, including atherosclerosis, heart failure, and stroke, has been known to increase by age in both women and men, linking to a number of factors such as apoptosis, oxidative stress, overall myocardial deterioration/degeneration, and inflammation (Benjamin et al., 2019).

The ER has a critical role in the normal function of the heart by providing the proper protein synthesis and folding in cardiomyocytes (Sozen et al., 2015; Sozen et al., 2020). Inflammation, metabolic disorder, and hypoxia occurring in CVD may trigger ER stress by affecting protein folding mechanisms (Diaz-Bulnes et al., 2019). Recent studies have reported increased levels of GRP78, XBP1s, CHOP, and ATF4 in cardiomyocytes of patients with heart failure (Ortega et al., 2014; Duan et al., 2015). Induced ER stress parameters might reflect involvement of UPR in promoting cardiac heart failure and hypertrophy, supporting the therapeutic value of ER stress targeting to reverse or reduce CVDs (Yao et al., 2017). Dual relationship between miRNA and ER stress in CVD is also reported (Maurel and Chevet, 2013). Knockdown of miR-30 in rat aorta vascular smooth cells and ventricular cells activated ER stress and induced ATF6, GRP78, caspase-12, and CHOP expressions (Chen et al., 2014). Additionally, miR-124 downregulation in cardiomyocytes reduced GRP78 and calreticulin (Bao et al., 2017), while miR-1283 inhibition in HUVEC cell line inhibited PERK/ATF4 activity and apoptosis, inflammation, and endothelial injury in heart tissue of mice (He et al., 2016). Another study determined the inhibitory effect of miR-133a against ER stress and apoptosis in rat cardiomyocyte (H9C2) cell line (Ren et al., 2019).

However, certain miRNAs may promote ER stress and predispose to CVDs (Ren et al., 2021). For instance, miR-103 in endothelial cells was highly expressed in atherosclerotic mice and upregulated inflammation and ER stress through miR-103–PTEN–MAPK axis (Jiang et al., 2020). Additionally, PERK-mediated miR-24 downregulation was reported to reduce mitochondrial dysfunction in heart failure (Shimizu et al., 2020).

### Cancer

The incidence of cancer in individuals over the age of 65 years has increased in most countries over the past decade. The risk of cancer in men and women increases approximately seven times with age compared to the young population in all types of cancer (Kumar et al., 2017). Extrinsic stresses and oncogenic activation exerted by the tumor environment are determined to induce UPR activation by increasing misfolded proteins. Numerous researchers have found the involvement of ER stress and UPR in many cancer types, including liver, lung, breast, glioma, and colorectal cancer (Shuda et al., 2003; Uramoto et al., 2005; Scriven et al., 2009; Auf et al., 2010; Jin et al., 2016). Cancer cells can evade prolonged UPR signal-mediated apoptosis and use UPR to stimulate metastasis and proliferation (Wang et al., 2010; Madden et al., 2019). However, UPR signaling might also limit the effectiveness of chemotherapy by generating chemoresistance (Chen et al., 2017; Salaroglio et al., 2017). Li X.-X. et al. (2017) found that silencing IRE1 in colon cancer cell line inhibited cell proliferation. Similarly, inhibition of IRE1 RNase activity decreased their proliferation capacity in breast cancer cells (Logue et al., 2018).

To date, certain miRNAs that play a role as an oncogene or tumor suppressor have been identified and characterized (Dragomir et al., 2018). miR-16 and miR-15, the first described cancer-related miRNAs, stimulate cancer development by decreasing anti-apoptotic BCL2 gene in chronic lymphocytic leukemia (Calin et al., 2002). Numerous studies using UPR-activated cancer cells have also determined miRNAs as a key modulator in cell death mechanisms. Therefore, IRE1 and ATF6 activation regulates tumor survival and progression by inhibiting miR-199a and miR-214 in human hepatocellular carcinoma cell lines, including HepG2 and SMMC-7721 (Duan et al., 2012). Additionally, most miRNAs in ER stress-induced cell death are determined to be involved in the PERK-eIF2α−CHOP pathway (Kim and Croce, 2021). Xu et al. (2019) showed that miR-451a overexpression activates apoptosis by inducing PERK/elF2α/ATF4/CHOP signaling in colorectal cancer cells, HCT116, and SW620. On the contrary, Huang et al. (2016) showed that ER stress-mediated induction miR-663 stimulates cell proliferation and reduces apoptosis in hepatocellular carcinoma cell.

## Conclusion

Impairments in ER-associated functions as well as alterations in miRNA levels in the aging process were reported to promote the progress of many age-related diseases. With more research conducted by basic and clinical scientists, more links will be established between the UPR and aging process. Recent advances arising from *in vitro* and *in vivo* studies have updated traditional principles of ER stress, changing our view of managing ER stress-related diseases (Table 3). In the light of the findings determining UPR/miRNA interaction in age-related diseases, miRNA therapy might be used to normalize gene expression alterations in the cells. So far, although the large number of studies using miRNAs greatly expanded our knowledge of their control over age-related diseases, their use in specific therapy is largely unknown. Despite these valuable findings, further studies are needed to determine mechanistic insights of ER stress and miRNA impairment as well as uncover additional miRNAs that might be considered as potential targets to open new doors in therapeutic approaches for aging and age-related diseases.

**TABLE 3:** Changing views on ER stress.

Past	Present
GRP78 mRNA or protein induction is a gold standard ER stress marker	GRP78 is a poor ER stress marker as it is a stable and abundant protein. Sensitive real-time markers, including phospho IRE1, phospho PERK, and activated ATF6, should be evaluated
eIF2α phosphorylation inhibits only 5′ methylguanylate (5me-G) cap-dependent mRNA translation	Phosphorylated eIF2α inhibits both cap-dependent and cap-independent mRNA translation
IRE1 RNase splices XBP1 mRNA, which encodes a potent transcription factor that activates expression of UPR target genes involved in ER proteostasis and cell pathophysiology	IRE1 RNase can also cleave mRNAs and precursor microRNAs leading to their degradation through regulated IRE1-dependent decay (RIDD), which modulates the protein folding load, cell metabolism, and apoptosis
miRNAs induce ER stress	miRNAs can also be processed during ER stress
IRE1, PERK, and ATF6 form three separate branches of the UPR	PERK generates a crosstalk with XBP1 by regulating the expression of various miRNAs

## Abbreviations

APP, amyloid β-precursor protein; ASK1, apoptosis signal-regulating kinase; ATF6, activating transcription factor 6; ATF4, activating transcription factor 4; CHOP, C/EBP homologous protein; CVD, cardiovascular disease; eIF2α, eukaryotic initiation factor 2α; FOXO1, forkhead box protein O 1; GADD34, growth arrest and DNA damage-inducible 34; GRP78, glucose-regulated protein 78; GRP94, glucose-regulated protein 94; IRE1, inositol-requiring enzyme; IRS1, insulin-receptor substrate; JNK, c-Jun amino-terminal kinase; NAFLD, nonalcoholic fatty liver disease; PDI, protein disulfide isomerase; PDIA6, protein disulfide isomerase family A member 6; PI3K, phosphoinositide-3-kinase regulatory subunit 1; PERK, protein kinase-like ER kinase; RIDD, regulated IRE1-dependent decay; TRAF2, tumor necrosis factor receptor-associated factor 2; UPR, unfolded protein response; XBP1, X-box binding protein 1.
